# Adaptation of the Main Peripheral Artery and Vein to Long Term Confinement (MARS 500)

**DOI:** 10.1371/journal.pone.0083063

**Published:** 2014-01-27

**Authors:** Philippe Arbeille, Romain Provost, Nicole Vincent, Andre Aubert

**Affiliations:** 1 Medicine Physiologie Spatiale (UMPS-CERCOM) University Hospital Trousseau, Tours, France; 2 CRIP Laboratoire d'informatique, Faculte de Medecine, Paris V, France; 3 University Hospital Gasthuisberg, O&N Lab. of Experimental Cardiology, KU Leuven, Leuven, Belgium; Bascom Palmer Eye Institute, University of Miami School of Medicine, United States of America

## Abstract

**Purpose:**

The objective was to check if 520 days in confinement (MARS 500), may affect the main peripheral arterial diameter and wall thickness and the main vein size.

**Method:**

Common carotid (CC) femoral artery (FA) portal vein (PV), jugular (JG), femoral vein (FV) and tibial vein were assessed during MARS 500 by echography, performed by the subjects. A hand free volumic echographic capture method and a delayed 3D reconstruction software developed by our lab were used for collecting and measuring the vascular parameters.

**Results:**

During the MARS 500 experiment the subjects performed 6 sessions among which 80% of the echographic data were of sufficient quality to be processed. No significant change was found for the Common carotid, Jugular vein, femoral artery, femoral vein, portal vein, and tibial vein diameter. CC and FA IMT (intima media thickness) were found significantly increased (14% to 28% P<0.05) in all the 6 subjects, during the confinement period and also at +2 days after the confinement period, but there was no significant difference 6 month later compare to pre MARS 500.

**Conclusion:**

The experiment confirmed that even untrained to performing echography the subjects were able to capture enough echographic data to reconstruct the vessel image from which the parameters were measured. The increase in both CC and FA IMT should be in relation with the stress generated by the confined environment or absence of solar radiation, as there was no change in gravity, temperature and air in the MARS 500 module, and minor changes in physical exercise and nutrition.

## Introduction

During spaceflight and simulated microgravity (bedrest) a human being is submitted to various environmental factors (microgravity, confinement, reduced physical activity, radiation..) supposed or proven to induce morphological and functional disturbances on most of the physiological system (cardio-vascular, bone, muscle, mental activity…) of which we do not know exactly the degree of reversibility especially for the cardiovascular system [1], [2], [3], [4].

The impact of real or simulated microgravity on the main physiological systems is already well documented, while those of the confinement are not because in spaceflight it is difficult to distinguish between the role of these 2 factors and in bedrest there is no real confinement.

Several studies of various duration were performed on healthy subjects to identify the impact of confinement and more precisely the role of the mental stress on the human being: ISEMSI 28days : [5] – EXEMSI 60days [6] – SFINCSS 110 d–240 days [7] - HUBES [8] - MARS 105days [9]. Heart rate decreased during confinement [9], [10], and oxydative stress increased. Renin, aldosteron, angiotensin, and arginin vasopressin increased [10]. Creatinin increased in relation with reduced water intake (hypo-hydration) and reduced physical activity [10]. Cell and humoral immunity were decreased [8]. Body weight and water loss and increased sodium were also reported [11]. Lastly a reduction in rapid memory acuity was mentioned [12].

The hypothesis of the MARS 500 experiment was that subjects in confinement may present vascular morphological and or functional changes in relation with the mental stress generated by the life in isolated condition or the environmental factors associated with the confined area.

The objective was to investigate during and after the confinement period peripheral (upper and lower part of the body) and central arteries and veins, as already done in other extreme environment like microgravity, bedrest, immersion (carotid, and femoral arteries – portal, femoral, and tibial, veins) during and after 520 day in confinement. A dedicated method based on echographic volume capture was used for the study to make the untrained and isolated subjects capable to capture images of the vessels without any assistance from outside.

## Method

### Population

6 healthy volunteers (Male, age: 26–38 years, weight 83.4+/−6 kg, height: 1.75+/−0.05 m) were selected for spending 520 days inside the MARS 500 module located at IMBP (Institute for Biological and Medical Problems) in Moscow. The protocol of this echographic study was approved by the ethical committee of the University Hospital Gasthuisberg, KU Leuven (Catholic University of Leuven), Leuven, Belgium and the ESA Medical Board (EAC, ESA Astronaut Center, Cologne, Germany) and the Institutional Review Board of the Institute for Medical and Biomedical Problems (IMBP) in Moscow, Russia and complied with all guidelines stated in the Declaration of Helsinki. ESA and IMBP were co-organizers of the Mars 500 study. Each subject was informed about the content and schedule of the experiment and signed the informed consent form.

During the whole duration the subjects had contact with the control centre via e mail only. A delay of up to 20 minutes in communication was built in during the expedition in order to mimic the real transmission delay between the MARS planet and earth.

During the 520 d confinement period the subject lived in the module of cylindrical section of 500 m3, and had scientific activity, moderate physical activity [7] and lived like on earth in the restricted area.

### Echographic capture

Before entering the module the subjects were trained 1 hour to perform on themselves a volumic capture of the organ/vessels they were supposed to investigate alone during the confinement period. The echographic investigation were always performed in seated position, because only this position allow self investigation by each volunteer. The subjects were taught to locate the echographic probe on top of the organ (according to a body cartography designed by our lab) and make a TILT movement with the probe body, the probe head remaining at the same place on the skin. All the echographic views collected during the TILT were store as a video file on a hard disc, and sent later on to the control center. Such maneuver does not require any practice in echography as the subject is not asked to perform a perfect long or short axis view of the vessel but only to scan the volume inside which the vessel is, by tilting the probe over 90 degree (+45° to −45° from the vertical to the skin). Thus the subject have just to be minimally trained and keep capable to locate the probe on top of the acoustic window of the organ.

In order to help the subject in locating the acoustic windows of each organ we identified the areas on the body with a high probability to find them. The position where to put the probe for visualizing the gall bladder/portal vein was found at the intersection of the right mammary and xyphoid lines. Around this point there is an area of approximately 8 cm diameter in which the probability to find the acoustic window of the gall bladder or portal vein is around 85%. (personal data obtained form 300 echographies). As soon as the subject saw a piece of either the gall bladder or the portal vein he performed the TILT and collected the echographic views. For the right kidney the acoustic window is located at the intersection of the right axilliary and xiphoid line. As previously, as soon as the subject saw part of the kidney he performed the TILT movement on the probe and recorded the echographic data. For the carotid they placed the probe at the bottom of the neck (contact with collar bone) in a transverse (horizontal) position and translated it slightly by 1 or 2 cm right to left until he centered the beating circle of the carotid in the middle of the image. Then they turned it by 90° while keeping the image of the vessel on the screen, then they Tilted the probe −45 to +45 degrees from the vertical to the skin. For the superficial femoral artery the probe was placed at the upper part of the thigh in a transversal orientation, when the beating circle of the artery appeared in the middle of the image the subject rotated the probe and Tilted it by −45 to +45 degrees from the vertical to the skin. During the Tilt (+45 to −45 degrees from the vertical to the skin) the subjects checked that the organ or vessel to capture was entirely scanned by the ultrasound beam and thus appeared and disappeared on the screen of the echograph. Tibial veins were investigated by putting the echographic probe body at the posterior face of the thigh parallel to the skin with the probe head in contact with the popliteal area. In this position we get a transverse view of the vessels of the popliteal area. In order to have a view of the tibial vein the subject was asked to translate the probe over 2 cm down to the calf.

The video files recorded during the Tilt of the probe were stored on a hard disk and sent to the control centre. At the control centre the video files were displayed as a series of images and reconstructed in a 3D space (Licence «Navigateur Echographique» EP 2 396 773), in which the expert could navigate with a virtual plan and select the view of the organ/vessel required for the measurement. Even if the vessel was scanned in oblique plans the 3D reconstructed volume allowed the scientist to find the long or short axis plan of the vessel required for the measurements. Thus the search of the appropriate vessel view was performed by the scientist after he received the files the subject never contributed to this phase.

### Measurement session

The subject performed echographic measurements in seated position as described above: 1 month pre MARS 500, during MARS 500 (3month, 6 m, 8 m, 11 m, 13 m, 16 m), post MARS 500 (+2 days, +6months). The data recorded at each session were sent to the scientist approximately 3 weeks later and processed at this time.

### Statistical analysis

Statistical analysis was performed using 1 way ANOVA (parameter being the effect of confinement) and validated with Tukey post hoc test (Sigma stat 3 Systat Software inc Chicago IL).

## Results

The subjects main cardiovascular parameters (Blood pressure and heart rate) did not show significant changes, as already shown previously during a preliminary study Mars105 (13) while body loss was moderate but significant for all subjects (mean: −8+/−7%.)

Because 20% of the echographic recording at the different session during the confinement period (3 months, 6 m, 8 m, 11 m, 13 m, 16 m) were not of sufficient quality to be processed in each of the 6 subjects we grouped the results in 2 session: 3–6 months and 11–16 months.

**Figure 1:**
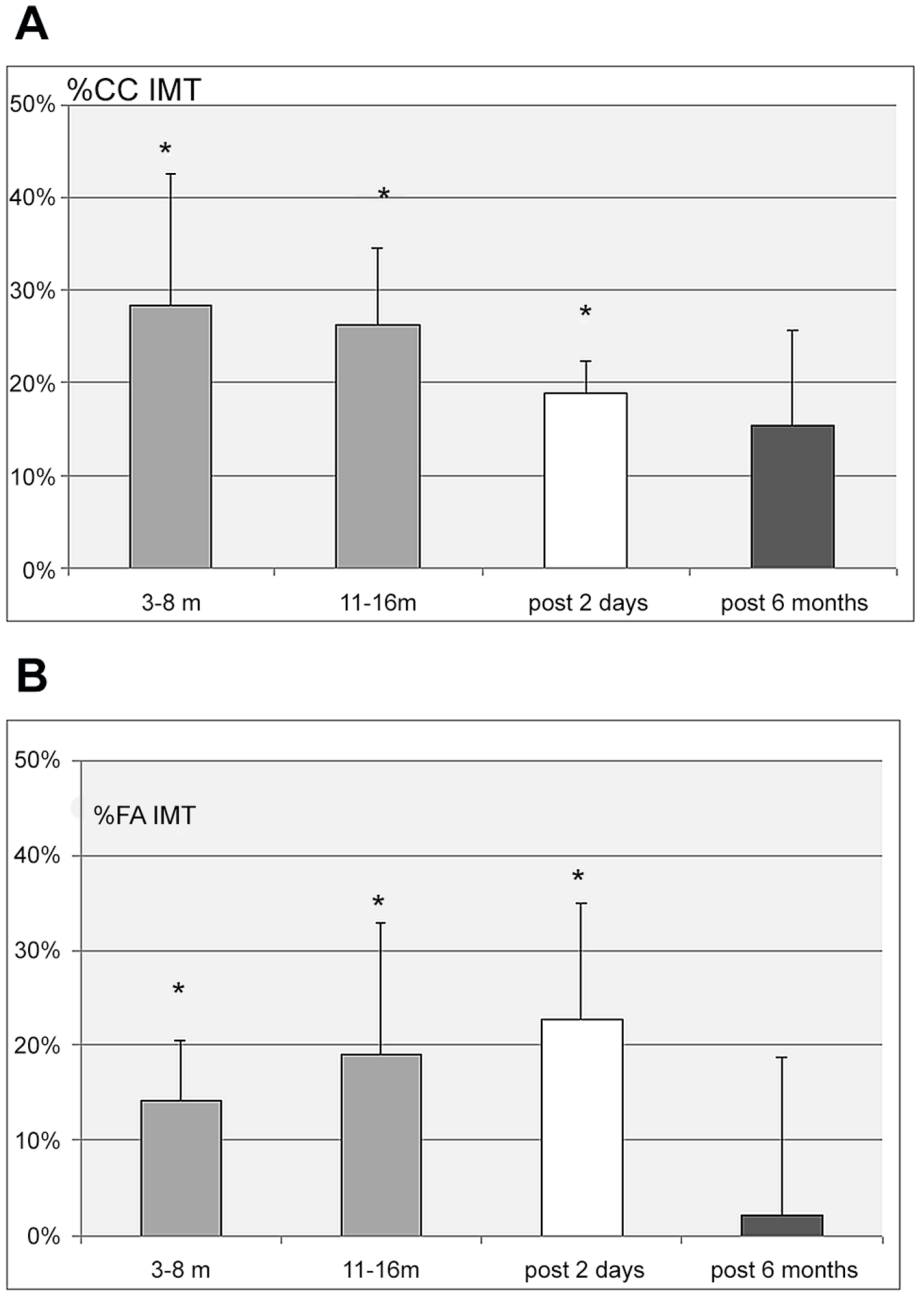
a: Percent change of the common carotid intima media thickness (CC IMT) from pre confinement values, during the confinement period (3 to 6 month, and 11 to 16 months) and 2 days and 6 months post confinement. (Mean value for the 6 subjects +/− SD - * P<0.05). b: Percent change of the superficial femoral artery intima media thickness (FA IMT) from pre confinement values, during the confinement period (3 to 6 month, and 11 to 16 months) and 2 days and 6 months post confinement. (Mean value for the 6 subjects +/− SD – * P<0.05).

**Figure 2:**
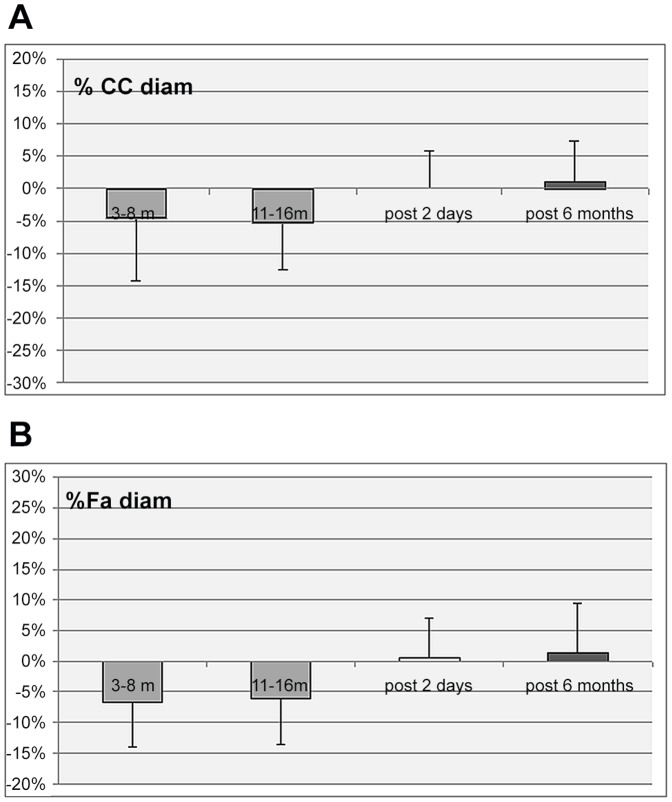
a: Percent change of the common carotid diameter (CC diam) from pre confinement values, during the confinement period (3 to 6 month, and 11 to 16 months) and 2 days and 6 months post confinement. (Mean value for the 6 subjects +/− SD - no significant change). b: Percent change of the superficial femoral artery diameter (FA diam) from pre confinement values, during the confinement period (3 to 6 month, and 11 to 16 months) and 2 days and 6 months post confinement. (Mean value for the 6 subjects +/− SD - no significant change).

**Figure 3:**
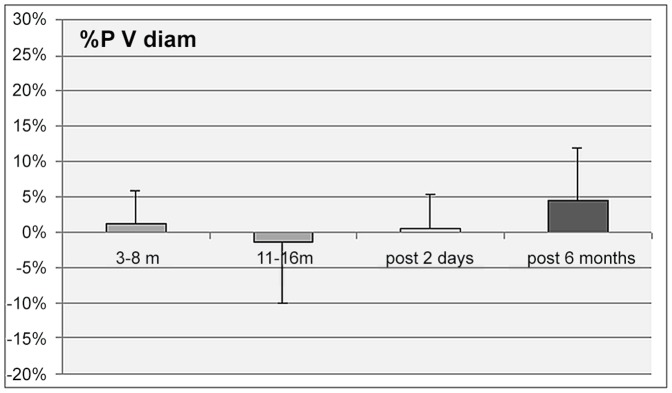
Percent change of the portal vein diameter (PV diam) from pre confinement values during the confinement period (3 to 6 month, and 11 to 16 months) and 2 days and 6 months post confinement. (Mean value for the 6 subjects +/− SD - no significant change.).

No significant changes were found for the common carotid, jugular vein, superficial femoral artery, femoral vein, portal vein, and tibial vein diameter or cross section area. There was a significant increase in intima media thickness (IMT) of the carotid and femoral arteries all along the MARS 500 confinement period and also at +2 days after the confinement period, but no significant difference 6 month later compared to pre MARS 500. (TABLE 1; Figure 1a). The portal vein, carotid and femoral data at rest prior to the confinement, at 3–8 m and 11–16 m of confinement and post confinement are presented on Table 1, as mean+/−SD and their change in % from base on figure 1–3.

**Table 1:** Portal vein diameter (PV diam) common carotid diameter and IMT (CC diam; CC IMT), superficial femoral artery diameter and IMT (FA diam; FA IMT) mean value and standart deviation (SD) averaged over the 6 subjects.

	PV diam (cm)	*SD*	CC diam (cm)	*SD*	CC IMT (cm)	*SD*	FA diam (cm)	*SD*	FA IMT (cm)	*SD*
pre	1,07	*0,14*	0,681	*0,056*	0,043	*0,003*	0,669	*0,038*	0,047	*0,010*
3–8 months	1,04	*0,07*	0,648	*0,050*	0,056*	*0,008*	0,626	*0,077*	0,053	*0,008*
11–16 m	1,05	*0,09*	0,644	*0,057*	0,055*	*0,004*	0,629	*0,062*	0,055	*0,009*
Post 2 days	1,08	*0,13*	0,679	*0,023*	0,051*	*0,003*	0,674	*0,060*	0,057	*0,007*
Post +6 m	1,12	*0,15*	0,687	*0,039*	0,050	*0,007*	0,679	*0,074*	0,048	*0,014*

* significant P<0.05.

CC IMT was found increased by 28%+/−14% (p<0.05) during the period 3 to 8 months, then by 26%+/−8% (p<0.05) during the period 11 to 16 months, and by 19%+/−3% (p<0.05) at +2 days after MARS 500. At 6 months CC IMT was not significantly different from pre MARS 500.

The superficial femoral artery IMT also increased significantly during MARS 500 period compared to pre MARS 500: 14%+/−6% at 3–8 months (p<0.05), 19%+/−14% at 11–16 months (p<0.05), and 23%+/−12% (p<0.05) at +2 days post MARS 500. Then it returned to pre MARS 500 level at 6 month (Figure 1b).

Consequently the ratio CC diam/CC IMT decreased significantly during MARS 500 (−24%+/−12% 3–8 months, and 11–16 months p<0.05) and at post 2 days (−16+/−4% p<0.05). On the other hand FA diam/FA IMT decreased (−21%+/−5% 3–8 months; −20%+/−9% 11–16 months) p<0.05) during MARS 500 period and decreased significantly (−17%+/−10% p<0.05) at 2 days post MARS 500.

## Discussion

For assessing the cardiovascular system during the confinement period with a delay of audio video communication of approximately +20 min it was impossible to assist the subjects from distance. Thus the subject were asked to perform a volumic capture of echographic images as described in the method section. Despite having been familiarized for only 1 h before the MARS 500 confinement period, they were able to capture appropriate volumes of echographic images containing the vessel at least in 80% of the sessions. This means that in 20% of the capture, the vessel was not entirely inside the volume scanned during the TILT, thus the scientist could not reconstruct the complete view of the vessel and make the measurements.

No significant morphological changes were identified in the main systemic and splanchnic circulation. The main peripheral arteries (carotid and femoral) and the portal and femoral and tibial vein diameters remained unchanged during and after the 500 day confinement period compare to pre.

Carotid and femoral results suggest that the main peripheral arterial flows remained stable as expected, because in absence of gravity change there would be no fluid redistribution between the main vascular compartment, nor a decrease in plasma volume as observed in bedrest or spaceflight [1], [4]. Moderate but significant body weight and plasma volume loss were reported in 28, 60 and 105 day confinement study [10], [11], [13] while in the present one despite the subjects reduced slightly their physical activity and changed their salt intake no body weight nor water content loss was reported [16]. Thus the vessel wall change measured during MARS 500 could not be related to significant hemodynamic changes. No significant change in the portal vein size supports the hypothesis that long term confinement did not induce any splanchnic flow volume change. Conversely during a 60 day bedrest where the subject lived in a confined area and remained in an anti-orthostatic position 24 h a day, a significant decrease in the portal vein area was measured in control (non exercising) subjects [14]. this observation suggests that the changes in portal vein area during head down bedrest were mainly related to the head wards fluid shift and absence of exercise which reduce plasma volume.

The femoral and tibial vein diameter did not increase which means that there was no abnormal blood pooling in the legs veins. On the other hand the jugular vein was not enlarged like in microgravity or head down bedrest [1], thus there was no venous stagnation at the cephalic level too.

No sign of orthostatic intolerance was observed at the end of the experiment, thus we can consider that the sympathetic and the distal neuro-vascular response to acute fluidshift (seated to stand up) were not affected as it is the case after a space flight or a bedrest.

Carotid and femoral IMT were found significantly increased during the whole confinement period and remained elevated at +2 days after the end of the confinement while it returned to basal level 6 months later. Such observation rises at least 3 hypothesis: (a) IMT increase should be part of a pathological process induced by confinement through the mental stress, or other metabolic processes, (b) IMT increase reveals an acceleration of the aging effect observed on healthy sedentary human across time, (c) IMT increase is part of the reversible adaptation of the cardiovascular system to the environment associated with the confinement like absence of solar radiation, nutritional regime, physical activity.

Simulated microgravity on rats (28 days in tail suspension) induced a cerebral artery wall thickness and cross-sectional area increase, whereas it induced a decrease in cross sectional area and vessel wall thickness at the mesenteric artery level. Moreover daily 1-h – hyper gravity fully prevented these changes in both kinds of arteries [15]. During tail suspension the animal was submitted to fluid shift towards the head and various stress related to the confinement, and the constraints due to the abnormal position. During MARS 500 only IMT was increased, while the subject was submitted to confinement stress induced but not to any gravitational change nor fluid shift. Conversely in simulated microgravity the animal was submitted to stress plus head ward fluid shift and both diameter and IMT increased. One may suggest that IMT and diameter increase should be related to different factors in these 2 cases.

Patients with cardiovascular risk factors and increased IMT have a higher risk to have cardiovascular events than those with normal IMT [17], [18]. Thus IMT increase is considered as a risk factor or a marker of aging at the vascular level like the increase in distal vascular resistance or hypertension. Nevertheless despite an increased IMT is considered as a risk factor associated with atheromatous lesion, no correlation was found between elevated IMT and presence of high degree of stenosis [18].

In diabetes patient with increased IMT the phosphodiesterase inhibitor cilostazol was found to reduce the IMT, while the IMT remained increased in patients not treated [19]. But in the MARS 500 study the subjects had a nutritional regime well calibrated which could not reasonably induce any metabolic disorder.

Stable arterial hypertension in young men was associated with signs of remodeling of common carotid artery walls (increased IMT) and increase of their rigidity [20]. Such population was closer to the MARS 500 one, than general cardiac patient as the subjects had no multiple cardiovascular risk factor or significant cardiovascular disease but the MARS 500 population did not show any increase in blood pressure.

In a normal population (without cardiovascular disease), the carotid **IMT** was found to increase with age and its determinants associated with age and gender [21]. In the case of MARS 500 the duration was not long enough to explain the IMT increase by an effect of aging, moreover the process was reversible.

As the MARS 500 volunteers had no cardiovascular or other pathology, or risk factors when entering the confinement habitat it is difficult to suggest a pathological mechanism for explaining the IMT increase. Moreover the diameter of the arteries concerned did not change at all.

Thus we suggest that the increase in IMT should be related to one or several environmental factors of the confinement habitat. There was no change in gravity, atmospheric pressure, oxygen pressure, and slight changes in nutrition or physical activity… compared to sedentary people not living in a confined environment. One may suggest that other factors like the isolation from the outside, the supposed outside risky environment itself, the absence of solar radiation could request an adaptation of some of the human body function and trigger metabolic processes.

An oxidative stress related to confinement could be a factor involved into the vascular changes observed during MARS 500. Oxidative stress is related to abnormal oxygen metabolism which produces nitric oxide and other element known to favor inflammatory reaction at the vascular level with increased IMT [22], [23]. Such hypothesis is supported by the results from a 105 d confinement experiment performed one year ago in the same facility as for MARS 500. This study reported an increase in oxidative stress with increase in oxi-hemoglobin, and decrease in some antioxidant defense [24].

Other studies reported that confinement induce mental (emotional) and physical stress that were found to disturb several cardiovascular target properties like arterial stiffness, endothelium properties, capillary permeability in relation with edema, or homodynamic parameters (mean arterial pressure ?), or parameters regulating cardiovascular status like autonomous nervous system, insulin resistance, increase cathecholamines, angiotensine II, nitric oxide [25]. In normal population longitudinal studies showed that social isolation in children was associated with higher cardiovascular risk factors when adults [26], and that social isolation in adult also increased cardiovascular risk factors [27]. Depression, anxiety, mental disorder (confusion) that can be induced by confinement are also considered as cardiovascular risk factors [28], [29]. Confinement was also found to reduce capacity to concentrate and increase the time needed to make a decision [30], [31]. Thus putting together several individuals of various personality may create tension or collaborative influence between the participant and generate mental stress [32]–[34], [37].

At last solar Ultra-violet rays induce the synthesis of vitamine D, and their decrease as observed in winter in northern country are responsible for a decrease in vitamine D [35], [36]. Such results suggest that the MARS 500 subject who were never exposed to solar radiation while exposed to calibrated artificial light may have suffered for a lack in vitamine D synthesis. Knowing that deficit in vitamine D in human was found associated with cardiovascular disease and significant carotid IMT increase [38] one may suggest that the increase in IMT should be related at least partially to the lack in Vitamine D in relation with absence of solar radiation.

### Limitations

This study is limited by small number of subjects (*n* = 6), which must be accepted given the limitation of crew members within the MARS 500 program and which is a general problem of space related research. Nevertheless the data show a clear influence on cardiovascular morphological alterations induced by confinement. Due to the limitations of access and experimental procedures within the MARS 500 facilities, it was not possible to obtain more physiological parameters.

## Conclusion

Confinement induces cardiovascular morphological alterations probably in relation with a metabolic process (and not with a physical one like microgravity) induced by the confinement and environmental factors among which the absence of solar radiation, knowing that the other environmental factor like gravity, atmospheric and oxygen pressure, nutrition, fluid intake, sleeping rate, and exercise activity were not different than in normal life. Nevertheless such suggestion have to be confirmed as it was difficult to get a quantification of these factors during the present MARS 500 experiment. Moreover after MARS 500 an additional and unexpected challenge will be to design appropriate counter measures for preventing the increase in IMT.
